# Von Economo Neurons – Primate-Specific or Commonplace in the Mammalian Brain?

**DOI:** 10.3389/fncir.2021.714611

**Published:** 2021-09-01

**Authors:** Ivan Banovac, Dora Sedmak, Miloš Judaš, Zdravko Petanjek

**Affiliations:** ^1^Department of Anatomy and Clinical Anatomy, University of Zagreb School of Medicine, Zagreb, Croatia; ^2^Croatian Institute for Brain Research and Center of Excellence for Basic, Clinical and Translational Neuroscience, University of Zagreb School of Medicine, Zagreb, Croatia

**Keywords:** von Economo neurons, human, primate brain, anterior cingulate cortex, frontoinsular cortex, cerebral cortex, pyramidal neuron

## Abstract

The pioneering work by von Economo in 1925 on the cytoarchitectonics of the cerebral cortex revealed a specialized and unique cell type in the adult human fronto-insular (FI) and anterior cingulate cortex (ACC). In modern studies, these neurons are termed von Economo neurons (VENs). In his work, von Economo described them as stick, rod or corkscrew cells because of their extremely elongated and relatively thin cell body clearly distinguishable from common oval or spindle-shaped infragranular principal neurons. Before von Economo, in 1899 Cajal depicted the unique somato-dendritic morphology of such cells with extremely elongated soma in the FI. However, although VENs are increasingly investigated, Cajal’s observation is still mainly being neglected. On Golgi staining in humans, VENs have a thick and long basal trunk with horizontally oriented terminal branching (basilar skirt) from where the axon arises. They are clearly distinguishable from a spectrum of modified pyramidal neurons found in infragranular layers, including oval or spindle-shaped principal neurons. Spindle-shaped cells with highly elongated cell body were also observed in the ACC of great apes, but despite similarities in soma shape, their dendritic and axonal morphology has still not been described in sufficient detail. Studies identifying VENs in non-human species are predominantly done on Nissl or anti-NeuN staining. In most of these studies, the dendritic and axonal morphology of the analyzed cells was not demonstrated and many of the cells found on Nissl or anti-NeuN staining had a cell body shape characteristic for common oval or spindle-shaped cells. Here we present an extensive literature overview on VENs, which demonstrates that human VENs are specialized elongated principal cells with unique somato-dendritic morphology found abundantly in the FI and ACC of the human brain. More research is needed to properly evaluate the presence of such specialized cells in other primates and non-primate species.

## Introduction

**FIGURE 1:**
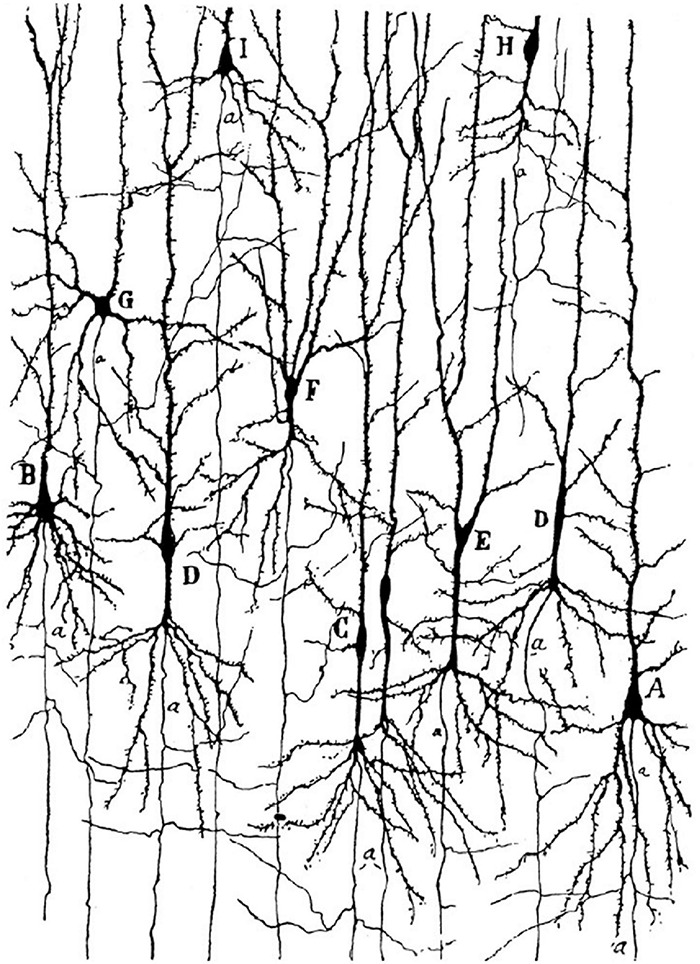
Golgi staining of the FI in a 1-month-old human girl. Note the von Economo neurons (VENs) marked by the letters C and D, and the very distant axon origin of these cells. Image acquired from Cajal (1899).

Von Economo neurons (VENs) is a term that was originally used to define a specialized type of principal cortical cell with specific morphology (Watson et al., 2006) and distinct areal and laminar distribution first described in detail by Constantin von Economo (1926). Using Nissl and Bielshowsky silver staining that visualize the cell body and proximal dendrites, von Economo noted cells with extremely elongated stick-like or corkscrew-like soma shape in the human cerebral cortex. These cells were grouped into cell clusters (usually 3–5 neurons) in layer Vb of the fronto-insular (FI) and anterior cingulate cortex (ACC). The shape was so unique that he first though they represented a pathological alteration (von Economo, 1918), but later he found them to be a unique cell type. Interestingly, it was Cajal (1899) who first noted the unique somato-dendritic and axonal morphology of these cells (Figure 1), two and a half decades before von Economo recognized them as a specialized cell type (*Spezialzellen*).

**FIGURE 2:**
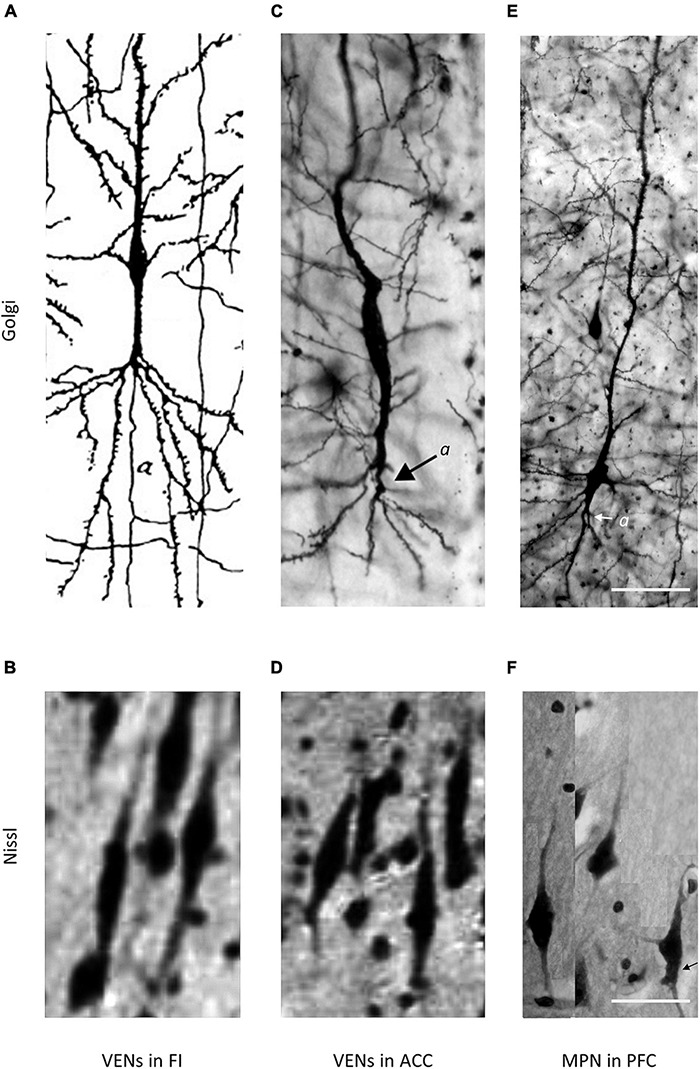
Comparison of VENs and common modified pyramidal neurons (MPN) in the fronto-insular (FI), anterior cingulate cortex (ACC), and PFC on Golgi and Nissl staining. Axons on Golgi staining are marked by “*a*”. The magnification for all microphotographs is indicated by the 50 μm scale bar in the lower right corner. **(A)** Drawing of a VEN in the FI of a 1-month-old human, Golgi staining. Image modified from Cajal (1899). Note the distant axon origin and the brush-like terminal branching of the prominent basal dendrite. **(B)** Microphotograph showing a cluster of VENs in the FI of an adult human, Nissl staining. Image modified from von Economo and Koskinas (1925). **(C)** Microphotograph of a VEN in the ACC of an adult human, Golgi staining. Image modified from Banovac et al. (2019). Note the distant axon origin and the brush-like terminal branching of the prominent basal dendrite. **(D)** Microphotograph showing a cluster of VENs in the ACC of an adult human, Nissl staining. Image modified from von Economo and Koskinas (1925). **(E)** Microphotograph of a common MPN with a spindle-shaped cell body found throughout the PFC, Golgi staining. Image modified from Banovac et al. (2019). Note the axon origin close to the cell body and the lack of the brush-like terminal branching typical for VENs. **(F)** Microphotograph showing several common MPNs with a spindle-shaped cell body found throughout the PFC, Nissl staining. Image modified from Banovac et al. (2019).

We would like to stress out that the specialized cells described by von Economo correspond to cells with unique dendritic morphology and axon origin already indicated by Cajal on Golgi staining (Figure 2). In the most comprehensive study of the cytoarchitectonics of the human cerebral cortex, von Economo determined that VENs were present abundantly and exclusively in layer Vb of the FI and ACC (von Economo and Koskinas, 1925). There is still no convincing evidence that such grouping of these specialized cells could be found in other cortical layers or regions in humans and there is no evidence at all that such specialized could be particularly abundant in any non-primate species.

Modern studies on VENs using Nissl and anti-NeuN staining that demonstrate only the cell body became the most prevalent in the field. Thus, VENs were predominantly identified based on their cell body shape (elongated fusiform soma) and found in regions other than the FI and ACC, and in species other than humans and non-human primates (Hof and van der Gucht, 2007; Hakeem et al., 2009; Butti et al., 2014; Raghanti et al., 2015). However, in most of these studies, the dendritic and axonal morphology of the cells defined as VENs was not demonstrated and Cajal’s initial descriptions were neglected.

Therefore, the central issue of this field of research is whether the definition of VENs (or lack thereof) in newer studies encompasses a significantly wider neuron population than the special cells defined by von Economo and described by Cajal. In this manuscript we present a detailed overview of classical and modern studies on VENs and discuss what characterizes the neurons initially defined by von Economo to be abundantly present in only two regions of the human cerebral cortex as a truly specialized and distinct sub-type of principal cell.

## Classification of Principal (Pyramidal) Neurons in the Cerebral Cortex

In order to define a certain type of neuron as a special cell, it is necessary to specify the criteria that separate such cells from the majority of other neurons. To understand if VENs have the required morphological features to be defined as a special cell type, we first give a brief description of the morphology of different classes of neurons within the cerebral cortex.

Based on their somato-dendritic morphology on Golgi staining, telencephalic cortical neurons can be classified into one of three groups: pyramidal cells, modified pyramidal cells, and non-pyramidal cells (Braak, 1980; Braak and Braak, 1985; Petanjek and Kostović, 1994a, b).

Non-pyramidal cells are a diverse morphological group of non-projection (local circuit) neurons, consisting of inhibitory (GABAergic) interneurons (Braak, 1980; Džaja et al., 2014; Hladnik et al., 2014) and excitatory (glutamatergic) spiny stellate cells (Okhotin, 2006). Unlike pyramidal and modified pyramidal cells, inhibitory interneurons give only a small number of very thin dendrites that lack spines and arise (and bifurcate immediately) from the cell body. Their axon can arise from any part of the cell body, including from the lateral side with a lateral course in the same cortical layer (Braak, 1980). In contrast to inhibitory interneurons, the dendritic morphology of spiny stellate cells bears more similarities to that of small pyramidal neurons found in neighboring cortical layers and during development these cells are presumed to retract their extracortical axon branch and apical dendrite (Meyer et al., 1989; Vercelli et al., 1992; Callaway and Borrell, 2011). Considering these morphological and developmental features, spiny stellate cells are classified by some authors as a special subtype of modified pyramidal cells (Braak, 1980).

Pyramidal cells are excitatory glutamatergic neurons that represent the main type of principal cells in the cerebral cortex. They are named based on their cell body shape, have a prominent apical dendrite (oriented toward the cortical surface) arising from the apex of the cell body, and a variable number of less prominent basal dendrites (oriented toward the white matter) that arise from the base of the cell body (Braak, 1980; Petanjek and Kostović, 1994a). According to Braak (1980), the basal dendrites arise “from the inferior tips of the pyramidal soma as usually slender processes which bifurcate repeatedly as they extend distally.” The dendrites of pyramidal neurons have numerous spines. The axon typically arises “from the base of the cell body by way of a broad cone-shaped axon hillock” and is oriented toward the white matter. Occasionally, the axon may arise from the most proximal part of a thick basal dendrite (Braak, 1980).

**FIGURE 3:**
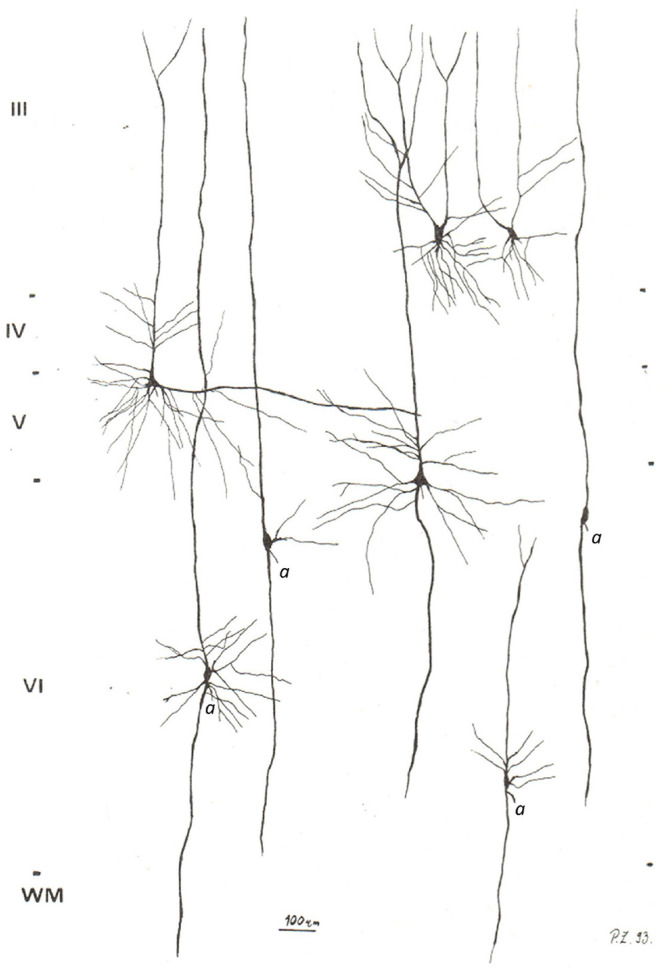
Camera lucida reconstructions of common modified pyramidal neurons in the PFC. The axons on spindle-shaped neurons are marked by “*a*”. Note that the axon of common modified pyramidal neurons arises either from the cell body or extremely close to the cell body, unlike the distant axon origin found in VENs. Also note that common modified pyramidal neurons lack the brush-like terminal branching typical for VENs. Image modified from Petanjek and Kostović (1994b).

Modified pyramidal cells is a term that describes a group of highly morphologically variable neurons with considerable morphological deviations from the typical appearance of the described pyramidal cells (Figure 3; Braak, 1980; Petanjek and Kostović, 1994b). To simplify, we will divide modified pyramidal cells into two groups: common modified pyramidal cells (found throughout the cerebral cortex and consisting of many different variants on a morphological spectrum) and specialized modified pyramidal cells (found only in specific cortical regions and layers and having unique morphological features).

Common modified pyramidal cells can have numerous somatic and dendritic morphological variations. Of particular importance are a group of common modified pyramidal cells with a fusiform cell body and a single prominent basal dendrite. This is characteristics of infragranular principal cells, such as layer VI principal cells with two prominent dendrites of approximately the same diameter and length (de Crinis, 1933; Braak, 1980). One of these dendrites is oriented toward the cortical surface (the “apical dendrite”), while the other can have various orientations, including toward the white matter (the “basal dendrite”). Many cells in layer VI have a typical bipolar organization with an oval cell body. The axon of these cells arises either directly from the cell body or as a side branch of the prominent basal dendrite, but still very close (up to 10 μm) to the base of the cell body. It is important to note that the common modified pyramidal neurons of infragranular layers represent a spectrum of morphological forms (Figure 3).

Specialized modified pyramidal cells have unique somato-dendritic and/or axonal morphology as well as specific areal and/or laminar distribution. Typical examples of such cells are giant Betz and Meynert cells found in layer V of the primary motor and primary visual cortex, respectively. Since the aforementioned cells described by von Economo and Cajal have both unique morphology and specific areal and laminar distribution, they can undoubtedly be classified as specialized modified pyramidal neurons. In subsequent chapters, we discuss that, according to von Economo’s own definition, the term “VENs” should principally be used to describe the specialized neurons abundantly found in layer V of the human FI and ACC. We also evaluate research that demonstrates the unique somato-dendritic and axon morphology of these cells as well as highlight the different descriptions of these cells in various research areas in the field.

## Defining the Terms – the Foundation Laid Out by Von Economo

In order to understand the early morphological descriptions of different cell types of the cerebral cortex, including VENs, it is necessary to define the terminology prevalently used in that time period. The most comprehensive overview of the morphological classification of the neurons of the cerebral cortex was given by von Economo and Koskinas (1925). They described the following three main groups of cells that make up the cerebral cortex: pyramidal, granule, and spindle (fusiform) cells (*Spindelzellen*). Besides these three fundamental cell types, von Economo and Koskinas also described different cellular types, characteristic of specific cortical regions, which they called special cells (*Spezialzellen*) of the cerebral cortex, such as the small horizontal and piriform cells of Cajal, the giant cells of Betz, the giant or solitary cells of Meynert, and the giant stellate cells. Here we give a brief description of the most relevant cell types, based on von Economo’s studies (von Economo and Koskinas, 1925; von Economo, 1927; von Economo and Triarhou, 2009).

Pyramidal cells are triangular-shaped, vertically elongated cells prominent in layers III and V. Their axon arises from the base of the soma (von Economo and Koskinas, 1925; Braak, 1980).

Spindle (fusiform) cells are long, spindle-shaped, vertically oriented and found in layer VI. Their soma has two poles that transform into long dendrites, which is why they are nowadays also sometimes referred to as bipolar fusiform cells. Note that spindle cells are characterized by a clear demarcation between the soma and the dendrites. The axon arises from the middle of the soma or from its lower end (von Economo and Koskinas, 1925; Braak, 1980).

**FIGURE 4:**
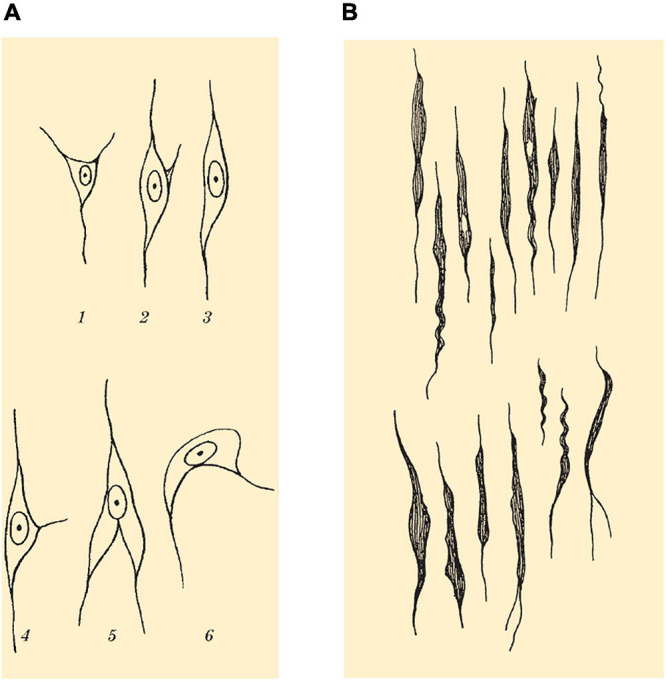
**(A)** Drawing showing spindle cells (*Spindelzellen*) of layer VI and morphological variants as seen on Nissl staining. **(B)** Drawing showing VENs (*Stab- und Korkzieherzellen*) of layer Vb found in the FI and ACC as seen on Nissl staining. Images modified from von Economo and Koskinas (1925).

Von Economo and Koskinas additionally described that pyramidal cells of layer V, and more rarely those of layer III, can assume a spindle-like form, which they called “spindle transformation” (*Verspindelung*) of the cells. Von Economo gave a comprehensive overview on the morphology of different spindle-shaped cells found in the cerebral cortex (Figure 4A; von Economo and Koskinas, 1925; von Economo, 1927; von Economo and Triarhou, 2009).

Note that von Economo does not describe spindle-like cells of layers III and V as spindle cells (*Spindelzellen*), but as spindle-transformed pyramidal cells – in more modern neuroanatomical terminology, we might describe such cells as a variant of common modified pyramidal neurons (Braak, 1980; Banovac et al., 2019, 2020).

Von Economo and Koskinas reserved the term special cells (*Spezialzellen*) for cells of not only peculiar morphology (differing from the typical morphology of the three main cell types), but also of distinct cortical and/or laminar distribution. Cells had to meet both criteria to be classified as special cells, meaning that cells having peculiar morphology was only a necessary, but not a sufficient criterion by itself. In fact, the key feature of special cells was the fact that they were abundantly present only in specific cortical regions and layers. Among these special cells, von Economo described a cell type found only in layer Vb of the FI and ACC. He referred to these cells as stick cells, rod cells or corkscrew cells (*Stabzellen, Stäbchenzellen*, or *Korkzieherzellen*) and gave a detailed description of their somatic morphology (Figure 4B; von Economo and Koskinas, 1925; von Economo, 1926, 1927; von Economo and Triarhou, 2009). Nowadays, we usually refer to these cells as VENs.

Von Economo acknowledged that spindle-shaped cells were briefly mentioned in the ACC by other authors before him (Hammarberg, 1895; Flechsig, 1897; Cajal, 1899; Nikitin, 1909; Marinescu, 1910). However, all of these authors described such cells as “spindle cells of layer V” in the ACC and did not recognize these cells as a type of special cell.

**FIGURE 5:**
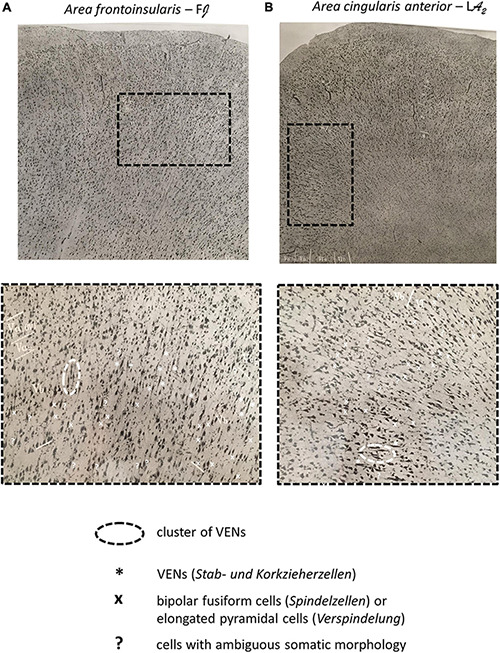
**(A)** Microphotograph of the dome of the *gyrus transversus insulae* (*Area frontoinsularis* – F*J*), Nissl staining. **(B)** Microphotograph of the dome of the *gyrus limbicus, regio anterior*; frontally and dorsally from the *genu corporis callosi* (*Area cingularis anterior* – L*A*_2_), Nissl staining. The enlarged panels show VENs in layer Vb of the FI and ACC as well as other spindle-shaped cells (*Spindelzellen*) and cells of ambiguous somatic morphology. Note that on Nissl staining, even in the FI and ACC, it is difficult to classify certain cells as either VENs or common modified pyramidal neurons (spindle cells, *Spindelzellen*). Also note the clusters of VENs in both regions as well as the relative predominance of VENs in the FI compared to the ACC. Images modified from von Economo and Koskinas (1925).

Von Economo and Koskinas described that the cells of the FI (Frontoinsular Area F*J*) were, in general, more elongated and spindle shaped. Layer VI spindle cells were moderately elongated compared to other cortical regions, while layer III pyramidal cells underwent spindle-transformation (*Verspindelung*). Nevertheless, it should be accentuated that layer Vb pyramidal cells were transformed to such an extreme degree that von Economo describes them as “absolutely spindle-shaped with regard to their vertical direction, and mostly form very long rod-like and also often spirally twined elements, extremely characteristic of this region, that we term rod and corkscrew cells” (Figure 5A; von Economo and Koskinas, 1925; von Economo, 1927; von Economo and Triarhou, 2009).

Unlike in the FI, von Economo describes no spindle transformation of the pyramidal cells in layer III and no overall cellular elongation in layer VI of the ACC. However, in layer Vb of the ACC (in the transition from area L*A*_2_ to L*A*_3_), von Economo describes that “a portion of the cells of sublayer Vb are conspicuously elongated, many being corkscrew-like” (Figure 5B). Besides these rod cells in sublayer Vb of area L*A*_3_, von Economo also described very slender, lancet-shaped pyramidal cells (von Economo and Koskinas, 1925; von Economo, 1927; von Economo and Triarhou, 2009).

It should be noted that von Economo demonstrated that in the FI (area F*J*), VENs were the dominant type of pyramidal cells in layer Vb, while in the ACC, VENs were not as dominant, but still abundant and clearly recognizable (von Economo and Koskinas, 1925; von Economo, 1927; von Economo and Triarhou, 2009).

From von Economo’s description, it is clear that he described VENs as a type of special cell in the human cortex found exclusively and abundantly in layer Vb of only two cortical regions – the FI and the ACC. It is also clear that von Economo considered these cells to be pyramidal neurons that underwent spindle-transformation (*Verspindelung*) to such an extreme degree not observed in any other part of the cortex. Because of their abundance in layer Vb of the FI and ACC and their peculiar cell body shape, these neurons (VENs) were completely different from spindle cells (*Spindelzellen*) found in layer VI and from typical spindle-transformed pyramidal cells usually found in layer V and occasionally in layer III throughout numerous cortical regions (von Economo and Koskinas, 1925; von Economo, 1927; von Economo and Triarhou, 2009).

Despite several claims that VENs were also described in the entorhinal cortex and/or subiculum (Butti et al., 2013; Cauda et al., 2014; González-Acosta et al., 2018), citing the work of Ngowyang (1936), this does not appear to have been stated in the paper itself. Namely, in this paper Ngowyang described in detail a different type of specialized cells (*Spezialzellen*) that he called fork cells (*Gabelzellen*). Moreover, Ngowyang (1936) studied cells in the Ammon’s horn and compared the morphology of fork cells to the morphology of a more ubiquitous cell type called *Umfassungszellen* (enveloping cells). Neither of these cell types morphologically resemble the rod- or corkscrew-shaped cells that we nowadays refer to as VENs.

## Historical Context of VEN Research

### Classical Studies Described VENs Only in Primates

**FIGURE 6:**
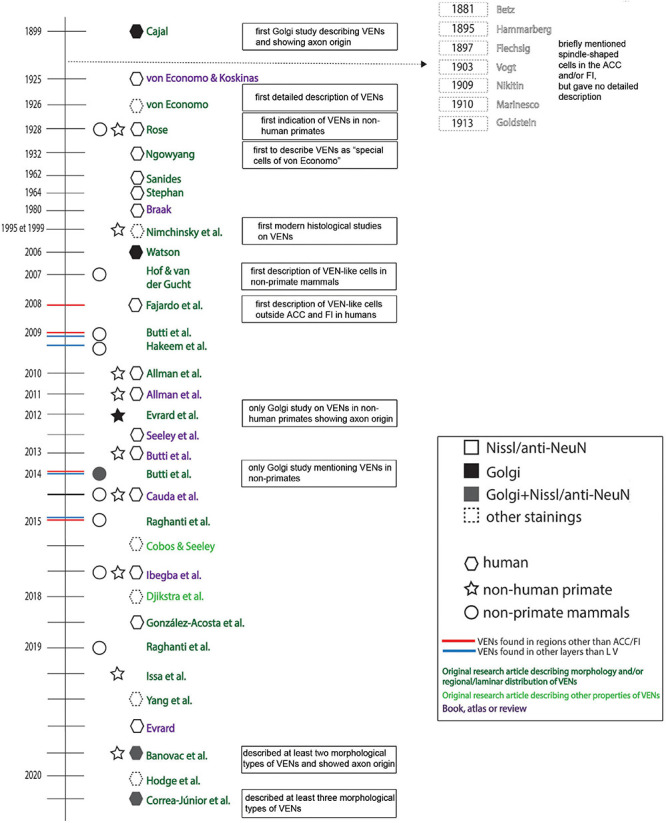
Historical timeline of the most relevant research and review papers on VENs in healthy/neurotypical brains.

A detailed historical timeline of the studies that described VENs in normotypical brains is given in Figure 6.

It is important to note that by 1867, spindle-shaped cells in the cerebral cortex have been described by Meynert (1867, 1868) as one of the three major cell populations (pyramidal, granule, and spindle cells). Several other pioneers of cytoarchitectonics also noted the existence of spindle-shaped cells in the human cerebral cortex, and many of them even briefly noted spindle-shaped cells in the ACC (Betz, 1881; Hammarberg, 1895; Flechsig, 1897; Vogt, 1903; Nikitin, 1909; Marinescu, 1910; Goldstein, 1913). These early descriptions failed to recognize the population of special cells found in the ACC (VENs) and still only referred to “spindle cells,” a term used for common fusiform neurons found throughout the cerebral cortex. None of these authors specified the extremely elongated rod or corkscrew cells in the ACC nor recognized that they differed from the common spindle cells of the cerebral cortex.

The first description of VENs’ unique somato-dendritic morphology using Golgi staining was given by Cajal (1899, 1995). However, it was not until von Economo (von Economo and Koskinas, 1925; von Economo, 1926, 1927) that VENs were recognized as a type of special cell with a highly specific areal and laminar distribution (layer Vb of the FI and ACC) in the human brain. Initially, von Economo (1918) observed VENs in “diseased brains” and briefly considered their normal morphology to represent a type of pathological alteration. He later revised his initial observations and gave by far the most comprehensive description of these cells in the human brain (von Economo and Koskinas, 1925; von Economo, 1926, 1927). Von Economo began using the terms “stick cells” and “corkscrew cells” to distinguish the special cells found in the FI and ACC from commonly found fusiform cells, which he referred to as “spindle cells.” Von Economo’s work on VENs remains relevant even for modern neuroanatomical research.

The first comparative studies briefly mentioning the existence of similar spindle-shaped cells in the same cortical regions of non-human primate species were conducted by Rose (1928a, b). The first author to refer to VENs as “the special cells of von Economo” (*von Economosche Spezialzellen*) was Ngowyang in 1932 (Ngowyang, 1932; Nieuwenhuys, 2012). Afterward, for decades most relevant neuroanatomical works mentioned VENs as special cells of the FI and ACC, as described by von Economo, but gave no new insight into the morphology or physiology of VENs (Sanides, 1962; Stephan, 1964, 1975; Braak, 1980). Until 1995, most authors referred to VENs as stick cells, corkscrew cells or the special cells described by von Economo. It should be noted that even though their extremely elongated soma was sometimes described as spindle-shaped, VENs were not referred to as “spindle cells.”

### Modern Studies and the Definition of VENs: Should All Neurons With a Spindle-Shaped Cell Body Be Defined as VENs?

The modern era of VENs research began in 1995 when Nimchinsky et al. (1995) carried out the first histological study focused on VENs using more contemporary methodology, including immunohistochemistry. This work was followed up by another study in 1999 (Nimchinsky et al., 1999) where a comprehensive comparative analysis of VENs between different primate species is given (see Figure 2 from Nimchinsky et al., 1999). It should be noted that from 1995 to 2006, VENs are predominantly referred to as “spindle cells” or “spindle neurons” (Nimchinsky et al., 1995, 1999; Allman et al., 2001). This is inconsistent with von Economo’s original terminology as von Economo reserved the term “spindle cells” (*Spindelzellen*) for a completely different class of cells typically found in layer VI throughout the cerebral cortex (von Economo and Koskinas, 1925; von Economo, 1927; von Economo and Triarhou, 2009).

The Golgi study by Watson et al. (2006) seems to be the first study to use the term VENs to describe spindle-shaped cells found in the FI and ACC, in an effort to reduce confusion associated with the terms “spindle cells” or “spindle neurons.” The term VENs has since then been predominantly used in most studies. However, whereas some studies used it to refer exclusively to von Economo’s special cells (stick cells or corkscrew cells), other studies used the term VEN to refer to any large, elongated spindle-shaped cell. This means that some authors appear to use the term VENs to describe the cells that von Economo called “spindle cells,” rather than just for the special cells (stick cells or corkscrew cells) he identified.

After Nimchinsky’s works, research on spindle-shaped cells in the cerebral cortex greatly expanded with comparative studies between different species (Table 1; Hof and van der Gucht, 2007; Butti et al., 2009, 2014; Hakeem et al., 2009; Allman et al., 2010, 2011b; Butti and Hof, 2010; Evrard et al., 2012; Raghanti et al., 2015, 2019; García-Cabezas et al., 2016; Evrard, 2019; Issa et al., 2019; Cabeen et al., 2020), studies on spindle-shaped cells in neuropathology or neurodivergent states (Table 2; Allman et al., 2005; Kaufman et al., 2008; Nitrini, 2008; Seeley, 2008; Simms et al., 2009; Brüne et al., 2010, 2011; Santos et al., 2011; Kim et al., 2012; Santillo et al., 2013; Santillo and Englund, 2014; Uppal et al., 2014; Gefen et al., 2015, 2018; Senatorov et al., 2015; Liu et al., 2016; Krause et al., 2017; Yang et al., 2017; Braak and Del Tredici, 2018; Fathy et al., 2018; Gami-Patel et al., 2019; Lin et al., 2019; Nana et al., 2019; Tan et al., 2019; Jacot-Descombes et al., 2020; Pasquini et al., 2020) and molecular studies (including immunohistochemical characterization and transcriptomics) (Fajardo et al., 2008; Stimpson et al., 2011; Cobos and Seeley, 2015; Dijkstra et al., 2018; Yang et al., 2019; Hodge et al., 2020). However, the amount of detailed morphological studies using Golgi staining (Watson et al., 2006; Banovac et al., 2019; Correa-Júnior et al., 2020), especially in non-human species (Evrard et al., 2012; Butti et al., 2014), remained remarkably low.

**TABLE 1:** Descriptions of von Economo neurons (VENs) in non-human species.

**Species**	**Regions in which VENs were described**	**Studies**
Great apes	ACC (Nissl)	Rose, 1928a, b; Nimchinsky et al., 1999; Allman et al., 2010; Issa et al., 2019; Evrard, 2019; Cabeen et al., 2020
Macaque monkey	ACC (Nissl) and FI (Nissl and Golgi)	Nimchinsky et al., 1999; Allman et al., 2010; Evrard et al., 2012; García-Cabezas et al., 2016; Evrard, 2019
Elephants	ACC and FI (Nissl)	Hakeem et al., 2009
Cetaceans	ACC, AI, and FP (Nissl)	Hof and van der Gucht, 2007; Butti et al., 2009; Raghanti et al., 2019
Manatee	ACC and insula (Nissl)	Butti and Hof, 2010
Pygmy hippopotamus	Frontal and temporal neo-cortex, primary visual and primary motor areas (Golgi and Nissl)	Butti and Hof, 2010; Butti et al., 2014
Artiodactyls and perissodactyls	frontal pole, ACC, anterior insula, occipital pole (Nissl)	Raghanti et al., 2015

**TABLE 2:** Alterations of VENs in various neuropathological or neurodivergent states.

**Neuropathology**	**Studies describing VEN alterations**
Alzheimer’s disease	Nimchinsky et al., 1995; Gefen et al., 2018; Tan et al., 2019
Autism spectrum disorder	Allman et al., 2005; Simms et al., 2009; Santos et al., 2011; Uppal et al., 2014
Amyotrophic lateral sclerosis	Braak and Del Tredici, 2018
Schizophrenia	Brüne et al., 2010; Brüne et al., 2011; Krause et al., 2017
Parkinson’s disease	Fathy et al., 2018
Frontotemporal dementia	Nitrini, 2008; Seeley, 2008; Kim et al., 2012; Santillo et al., 2013; Santillo and Englund, 2014; Yang et al., 2017; Gami-Patel et al., 2019; Lin et al., 2019; Nana et al., 2019; Tan et al., 2019; Pasquini et al., 2020
Agenesis of the corpus callosum	Kaufman et al., 2008
Alcoholism	Senatorov et al., 2015
Higher memory capacity in advanced old age	Gefen et al., 2015
Familial dysautonomia	Jacot-Descombes et al., 2020

Moreover, there are still no satisfactory functional studies on VENs that would correlate their unique morphology and cortical/laminar distribution to their role in the human brain, even though several papers speculated on the functional, clinical, and evolutionary relevance of VENs (Allman et al., 2001, 2011a; Watson and Allman, 2007; Seeley et al., 2012; Butti et al., 2013; Cauda et al., 2013, 2014; Ibegbu et al., 2015; Evrard, 2018; Bruton, 2021). In addition, studies that focused on molecular profiling of VENs yielded not a single specific marker that could be used to identify these cells without relying on morphological descriptions. Nevertheless, such studies revealed that VENs exhibit biochemical and transcription factors typical for glutamatergic projection neurons: SMI-32 (Nimchinsky et al., 1995), MAP2 (Fajardo et al., 2008), DISC1 (Allman et al., 2010), ATF3, IL4Ra, NMB (Stimpson et al., 2011), FEZF2, CTIP (Cobos and Seeley, 2015), VMAT2, GABRQ, and ADRA1A (Dijkstra et al., 2018). The aforementioned demonstrates that the molecular characterization of VENs is still not completed and it is likely that only a combination of molecular markers and morphological descriptors will provide means for reliable detection of these cells.

## New Challenges Arise in VEN Research

Molecular studies so far confirmed that VENs are a type of projection neuron, yet these studies yielded no specific maker for VENs. This means that reliable identification of these cells is still based on morphology and cortical/laminar distribution, though it seems the criteria applied in newer studies may deviate from von Economo’s original description. In the following paragraphs we present the different definitions of VENs applied in prominent papers as well as point out the somewhat conflicting findings of some of these studies.

The comparative studies by Butti et al. (2014) and Raghanti et al. (2015) applied only the morphological criterion of cells being large spindle-shaped neurons to classify them as VENs. These studies did not apply the requirement by von Economo that special cells ought to have specific cortical and laminar distribution as well. Studies on human brains by Fajardo et al. (2008) and González-Acosta et al. (2018) gave more importance to the morphological criterion, but still partly implemented the criteria concerning cortical and laminar distribution. Finally, studies in primates by Nimchinsky et al. (1999); Allman et al. (2010) and Evrard et al. (2012) described the analyzed cells slightly differently, though the way these cells were defined by Nimchinsky et al. (1999) appeared to be closest to von Economos’ original description.

The studies by Fajardo et al. (2008) and González-Acosta et al. (2018), both using anti-NeuN staining, claimed to have found VENs in human Brodmann areas (BA) 9 and 10, respectively. Interestingly, Fajardo et al. (2008) claimed in their study that they found no VENs in BA10 in none of the eight human subjects they analyzed, while González-Acosta et al. (2018) claimed to have found VENs in BA10 in all five human subjects they analyzed. Both studies acknowledged that the cells they described as VENs appeared to be far less abundant in these regions than the VENs in the FI and ACC (Fajardo et al., 2008; González-Acosta et al., 2018).

In the study by Nimchinsky et al. (1999), VENs were comprehensively analysed in the ACC of 28 different primate species using Nissl staining (Nimchinsky et al., 1999). According to this study, VENs appeared to be most numerous in humans (*Homo sapiens*), bonobos (*Pan paniscus*), and common chimpanzees (*Pan troglodytes*), less numerous, but still frequent in gorillas (*Gorilla gorilla gorilla*) and rare in orangutans (*Pongo pygmaeus*). No VENs were found in the ACC of any of the other 23 primate species analysed, including rhesus (*Macaca mulatta*) and cynomolgus monkeys (*Macaca fascicularis*) (see Table 1 in Nimchinsky et al., 1999). Over a decade later, these findings were almost entirely confirmed by Allman et al. (2010) using Nissl in the FI as well as the ACC (see Table 1 in Allman et al., 2010). Nevertheless, this study also claimed to have found VENs in layers other than layer V, which is not concordant with the study by Nimchinsky et al. (1999) that found VENs only in layer Vb (for a comparison of laminar distributions see Figure 3 in Nimchinsky et al., 1999 and Figure 3 in Allman et al., 2010). In contrast to both studies, the study by Evrard et al. (2012), found VENs to be abundant on Nissl staining in the agranular anterior insula, and somewhat scarcer, but still present in the ACC of both the rhesus and cynomolgus monkeys. Furthermore, Evrard et al. (2012) mentioned the presence of isolated VENs in BA10 and BA14. Interestingly, in this study, VENs were once again found only in layer Vb (see Figure 1F in Evrard et al., 2012).

The comparative studies by Butti et al. (2014) and Raghanti et al. (2015) claimed to have found VENs in numerous cortical areas of various mammalian species and even in layer III, though they described them as less numerous and more rarely found in layer III than in layer V. This is conspicuously similar to von Economo’s description of the spindle-transformation (*Verspindelung*) of pyramidal cells, which he described as occurring in layer V and occasionally in layer III in various cortical regions. However, it is important to note that von Economo did not consider such spindle-transformed pyramidal cells found throughout the cerebral cortex to be a type of special cell (*Spezialzellen*) (von Economo and Koskinas, 1925; von Economo, 1927; von Economo and Triarhou, 2009). It would, therefore, be an interesting point of discussion whether such ubiquitous cells found throughout the cerebral cortex should be referred to as VENs.

For descriptions in the human cerebral cortex, it seems more appropriate to reserve the term VENs for the special cells (rod or corkscrew cells) abundant in layer Vb of the FI and ACC. For comparative studies, it is reasonable to question how rigorously von Economo’s definition and Cajal’s description can or should be applied in non-human animal species. It is likely that VENs in other species have certain different or unique characteristics when compared to humans and this should be taken into account when conducting comparative studies. However, using a severely watered-down definition of VENs also does not seem productive in the further discourse on these cells, because it could lead to classifying any large spindle-shaped cell as a VEN. Finding the appropriate balance between straying too far away from the original descriptions of VENs and allowing for appropriate level of variety in non-human species is imperative in future comparative studies to advance the field. Expanding the methodology used in such studies (using Golgi, intracellular, or other molecular stainings) would be a step forward in resolving these issues.

## Morphological Criteria – Dendritic Morphology and Position of Axon Origin – Are Still Necessary for Reliable and Reproducible Identification of VENs

### Golgi Studies Analyzing VENs

**FIGURE 7:**
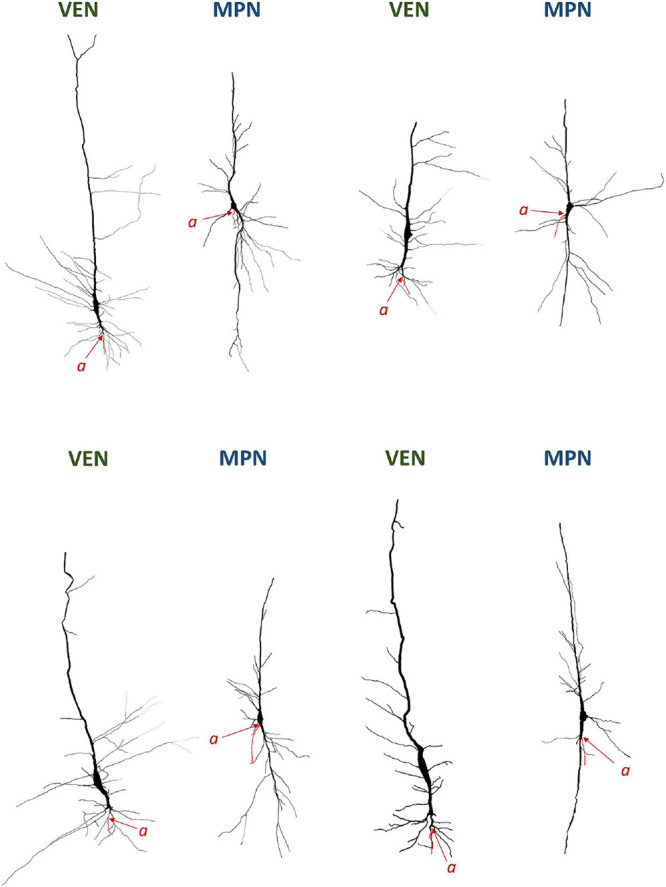
Neurolucida 3D reconstructions of VENs and common modified pyramidal neurons (MPN) found in the human cerebral cortex. The axon origin is marked by “*a*” and the axon is traced in red. Note the differences in axon origin between VENs and common MPNs as well as the brush-like terminal branching of the basal dendrite in VENs that is not present in common MPNs. Image modified from Banovac et al. (2019).

Since Cajal, only three Golgi studies on VENs were carried out in humans (Watson et al., 2006; Banovac et al., 2019; Correa-Júnior et al., 2020), and only two were carried out in non-human species (Evrard et al., 2012; Butti et al., 2014; Table 3). Out of those studies, only three (Cajal, 1899; Evrard et al., 2012; Banovac et al., 2019) demonstrated VENs’ axon origin, which appears to be the most distinct morphological feature of these neurons (Figure 7). All three of these studies were done in primates and in all of them, the axon of VENs was shown to originate far away (at least 100 μm) from the cell nucleus. The axon arose from either the basal dendrite or basal stem, not from the cell body as is the case for almost all other pyramidal and bipolar fusiform cells (for a detailed description of the somato-dendritic and axon morphology of VENs, see Banovac et al., 2019). Indeed, such an axon origin appears to be almost unique to the rod and corkscrews cells of layer Vb of the FI and ACC, and is found in cells of other cortical regions extremely rarely, at least in the primate brain (Braak, 1980). Due to the lack of detailed morphological studies in non-primate species, it is impossible to conclude whether this perhaps defining morphological feature is present in VEN-like cells observed in other species.

**TABLE 3:** Studies visualizing VENs using Golgi staining.

**Studies**	**Subjects used for Golgi staining**	**Region and cortical layer in which VENs were analyzed**	**Golgi staining method**	**VEN soma morphology**	**VEN apical dendrite/stem morphology**	**VEN basal dendrite/stem morphology**	**VEN axon morphology**
Cajal, 1899	1-month-old human female	FI; layer V	Classic Golgi staining	Spindle-like	Prominent with gradual decrease in thickness	Prominent, ends with a dendritic tuft (basilar skirt)	Arises from basal stem/dendrite
Watson et al., 2006	23-year-old human male	ACC and FI; layer V	Modified Golgi technique	Large and elongated, clear demarcation between soma and dendrites is visible in some neurons	Prominent, no additional dendrites or branching for a half-soma’s distance along the length of the proximal dendrites	Prominent, no additional dendrites or branching for a half-soma’s distance along the length of the proximal dendrites, most neurons shown lack a basilar skirt	Not shown
Evrard et al., 2012	One rhesus macaque	Anterior insula; layer Vb	Rapid Golgi-Cox	Spindle-like	Branches distally into several thinner spiny dendrites	Branches into thinner spiny dendrites in layer VI, visible basilar skirt	Arises from basal dendrite
Butti et al., 2014	One female pygmy hippopotamus	ACC, frontal magnocellular cortex, lateral gyrus*; layer V**	Modified rapid Golgi	Stout, sometimes slender	Almost as thick as the soma	Almost as thick as the soma, occasionally dividing into two branches with a hint of a basilar skirt	Not shown
Banovac et al., 2019	5 adult human male subjects	ACC; layer Vb	Rapid Golgi and Golgi-Cox	Large spindle-like, stick-like or corkscrew-like, on most neurons there is no clear demarcation between soma and dendrites	Very thick origin (up to 8 μm), very gradual decrease in thickness	Constant or very gradual decrease in thickness, low bifurcation degree, but high arborization (brush-like basilar skirt)	Arises from end of basal stem/dendrite
Correa-Júnior et al., 2020	4 adult human subjects (two male and two female)	ACC; layer Vb	“Single-section” Golgi	Large, elongated and spindle-shaped	Prominent with gradual decrease in thickness	Prominent with gradual decrease in thickness, low bifurcation degree, level of arborization depends on subtype (basilar skirt present in some neurons)	Not shown

*Other cortical areas were also analyzed, but only using Nissl staining or immunohistochemistry (frontopolar, temporal, parietal, and occipital cortex) and VENs were claimed to be found in all the analyzed cortical regions.

**VENs were claimed to occasionally have been found in the deep layer III of numerous cortical areas, but they were still most abundant in layer V and shown on Golgi only in layer V.

### Position of Axon Origin in VENs

It is interesting to note that in half of the Golgi studies on VENs, authors devoted little or no attention to the axon origin. All neurons demonstrated by Watson et al. (see Figures 1, 2, 4 from Watson et al., 2006) and Butti et al. (see Figures 13–15 from Butti et al., 2014), and some neurons demonstrated by Correa-Júnior et al. (see Figure 4 from Correa-Júnior et al., 2020) lack the typical dendritic morphology demonstrated by Cajal and in none of these studies was the axon origin identified or described. Some of the depicted neurons in these studies had a morphology similar to oval bipolar modified pyramidal neurons typical for layers V and VI of the cerebral cortex (Figure 3), rather than von Economo’s specialized cells. Nevertheless, a recent study referred in detail to Cajal’s original Golgi description (Figure 1), demonstrated the axon origin (Figures 2C, 7) and gave a comprehensive description of VENs’ dendritic morphology in humans consistent with Cajal’s initial description (Banovac et al., 2019). Furthermore, Evrard et al. (see Figure 2A from Evrard et al., 2012) demonstrated the axon origin of a single macaque VEN, which was in line with Cajal’s descriptions, even though the authors themselves put little emphasis on this morphological detail.

**FIGURE 8:**
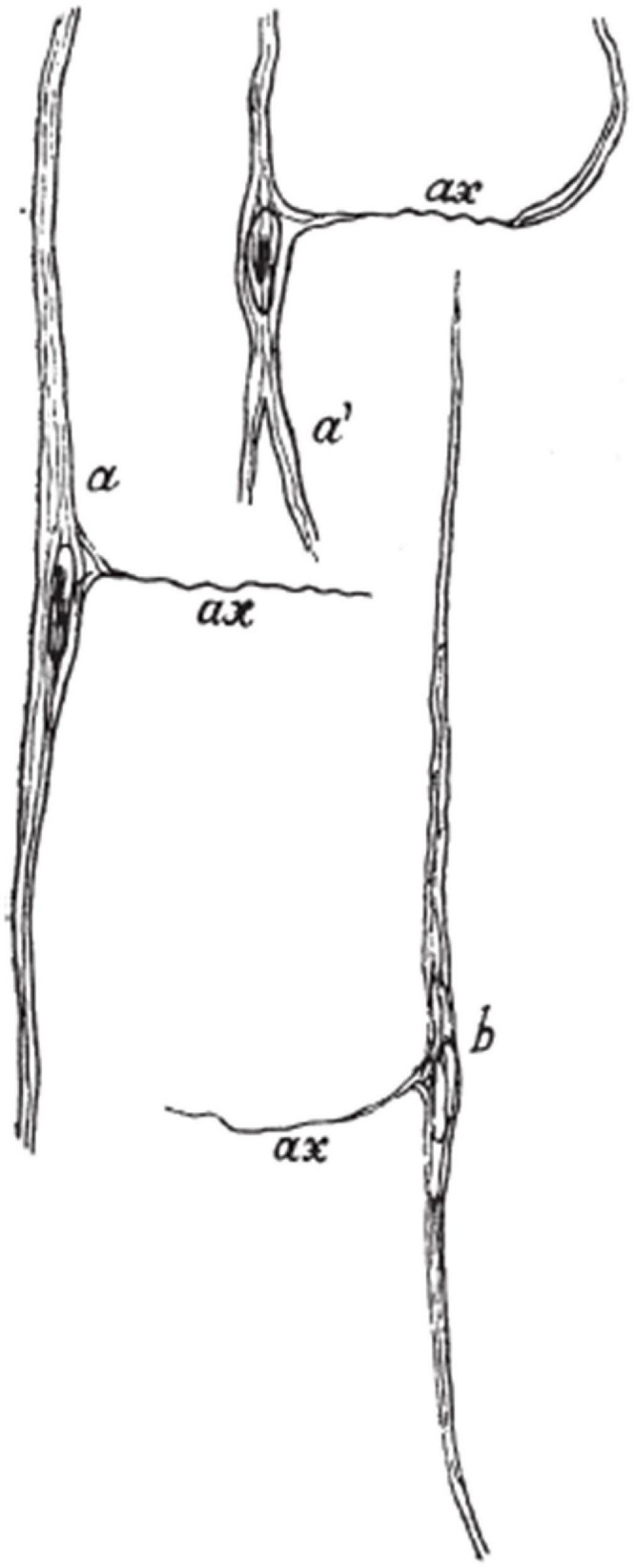
Drawing showing VENs as seen on Bielschowsky silver staining. Note the peculiar depiction of the axon arising laterally from the cell body, differing from all other studies that demonstrated the axon origin of VENs. Image acquired from von Economo (1926).

It is worth discussing that von Economo himself also described the axon of VENs using Bielschowsky silver staining. Interestingly, using this staining method, von Economo found no collateral dendrites arising from the soma or arborization of the apical or basal dendrites, but he described the axon of VENs as arising laterally from the soma, almost perpendicular to the soma’s orientation (Figure 8). Von Economo also described that the axon appeared to stay in the same layer as the cell it arose from von Economo (1926). This description is in stark contrast with all Golgi studies on VENs that showed the axon arising from the basal stem or basal dendrite and likely entering the white matter (Cajal, 1899; Evrard et al., 2012; Banovac et al., 2019). Furthermore, VENs having an axon that appears to stay in the same layer seems to be in contradiction with numerous studies confirming that VENs are projection neurons (Nimchinsky et al., 1995; Fajardo et al., 2008; Cobos and Seeley, 2015; Banovac et al., 2019; Hodge et al., 2020). It is possible that what von Economo visualized was actually a dendrite as most Golgi studies have shown that VENs have numerous thin dendrites arising from their cell bodies (Cajal, 1899; Banovac et al., 2019; Correa-Júnior et al., 2020). On modified pyramidal neurons it is common that a single, thin and long dendrite, sparsely populated with spines, arises from the middle side of the cell body, forming a third cell body angle (Braak, 1980; Petanjek and Kostović, 1994b). This dendrite has the same branching pattern as the process von Economo considered to be an axon, strongly supporting the view that the described process was actually a dendrite. Methodological differences between Bielschowsky silver and Golgi staining might explain the different interpretations between von Economo and other authors.

### Unique Dendritic Topology and Functional Properties of VENs in Humans

It should be noted that, even though the axon origin of VENs is probably their most defining characteristic (at least in humans), the morphology of the basal dendrite is also a distinct characteristic of human VENs. The prominent basal dendrite of VENs is almost as thick as the cell body and its thickness remains constant for most of its course before terminally branching into numerous thinner dendrites in a brush-like manner. This characteristic branching of the basal dendrite is referred to as a basilar skirt or basal dendritic tuft and is not described in other types of pyramidal or modified pyramidal neurons (Braak, 1980; Nimchinsky et al., 1995; Banovac et al., 2019; Correa-Júnior et al., 2020).

Finally, the overall antenna-like dendritic structure of VENs combined with a distant axon origin may have interesting functional implications. Studies so far have only speculated on the potential electrophysiological properties and general connectivity of VENs, however, most authors seem to agree that VENs ought to have some kind of highly specialized function in the cerebral cortex (von Economo and Koskinas, 1925; Nimchinsky et al., 1995; Watson and Allman, 2007; Stimpson et al., 2011; Seeley et al., 2012; Banovac et al., 2019). This is further supported by the fact that VENs are abundantly present in only two cortical regions in primates, the FI and ACC. Newer research suggests that the FI and ACC are part of the limbic system and may be functionally connected (Gu et al., 2010). The FI has been considered a sensory component of the limbic system responsible for polymodal sensory integration and representation of visceral responses (Critchley, 2004; Critchley et al., 2004). The ACC is considered a motor component of the limbic system involved in regulation of behavior, emotion, pain, and social interaction (Davis et al., 1994; Rainville et al., 1997; Cohen et al., 1999, 2001; Rudebeck et al., 2006).

For now, based on available data and without defined molecular markers specific for VENs, it seems prudent to exercise caution when identifying VENs in species and cortical regions/layers where VENs were previously not described. For research in humans and perhaps certain non-human primates, VENs may be reliably identified using only somatic staining, such as Nissl or anti-NeuN, provided the cells are restricted to layer Vb of the FI and ACC. Even in primates, identification of VENs outside these specific regions may benefit from using additional methods, such as (but not necessarily limited to) demonstrating the dendritic and axonal morphology using Golgi staining or retrograde intracellular injection (Elston et al., 2005a, b). Taking into account the available data, a set of morphological criteria for identification of VENs by combining Golgi and Nissl staining can be defined (Table 4; for a more detailed comparison of VENs and common spindle-shaped modified pyramidal neurons see Table 2 in Banovac et al., 2019). Combining multiple methods for identifying VENs and using clearer identification criteria should be a priority when attempting to confirm the presence of VENs in non-primate species.

**TABLE 4:** Morphological criteria for identifying VENs (adapted and modified from Banovac et al., 2019).

**Morphological feature**	**Characteristics of VENs**	**Recommended staining method for optimal assessment**
Apical process	Very thick origin (up to 8 μm), very gradual decrease in thickness	Rapid Golgi, Golgi-Cox, or other Golgi modifications that result in quality impregnation of the dendritic tree (possible alternative: intracellular dye injections)
Basal tree bifurcation degree	Low	
Basal process thickness	Constant or very gradual decrease	
Basal process arborization	High, brush-like (basilar skirt)	
Axon origin	End of basal stem	Rapid Golgi or other Golgi modifications that clearly visualize axon origin (possible alternative: intracellular dye injections)
Cell body shape	Long (50–120 μm) and stick-shaped (5–10 μm wide), preferably with clear regional and laminar specificity	Nissl, Golgi, or intracellular dye injections (possible alternatives: NeuN, SMI-32)

## Discussion

### VENs Are Specialized Cells That Represent a Distinct Population of Modified Pyramidal Cells

In mammalian species with a well-developed layer VI, throughout the cerebral cortex, layers V and VI are predominantly populated by oval or fusiform principal neurons with a vertical orientation toward the pia mater. The functional implications of this “spindle transformation” of principal neurons are still unclear and research on the density and distribution of spindle-shaped neurons in different species, cortical regions and layers is necessary for understanding the importance of this process. A limiting factor in such research is a lack of standardized classification, which would make identifying cells as spindle cells and differentiating common spindle cells from VENs more consistent. In some studies, authors described only cells lacking a clear demarcation between the soma and the main dendrites as VENs (see Figure 4 from Nimchinsky et al., 1995; Figure 1 from Nimchinsky et al., 1999; Figures 15, 16 from Hof and van der Gucht, 2007; Figures 1, 2 from Fajardo et al., 2008; Figures 1D, 2 from Evrard et al., 2012; Figure 16 from Butti et al., 2014; Figures 3–5 from Raghanti et al., 2015; Figures 2C,D from González-Acosta et al., 2018), while in others even oval bipolar neurons with a clear demarcation were described as VENs (see: Figure 4 from Watson et al., 2006; Figure 1A from Hakeem et al., 2009; Figures 4A, 5F from Butti et al., 2009). Some studies also considered cell body size when differentiating VENs from common spindle cells (Butti et al., 2009; Evrard et al., 2012), while others considered laminar/areal distribution (Watson et al., 2006; González-Acosta et al., 2018). Furthermore, most research so far, especially in non-primate mammals, has been done using methods that visualize primarily the cell body with the beginning of the main dendrites, without defining the molecular profile of the cells or displaying their dendritic and axonal morphology (including the presence/absence of dendritic spines).

The recent use of the term von Economo neuron (VEN) when referring to commonly found spindle-shaped cells in the cerebral cortex has caused further confusion in research on fusiform cells and spindle transformation, even though von Economo in his writings accentuated a special cell type, which he called stick or corkscrew cells. Von Economo also explicitly stated that these special cells with a stick- or corkscrew-shaped cell body were clearly distinguishable from other spindle-shaped cells found throughout the cerebral cortex. It is clear that the special cells described by von Economo had a distinct areal and laminar distribution in the human brain. Cajal’s description indicated that these special cells had unique dendritic and axonal morphology that was not present in any other cell type in the cortex. Although Cajal clearly demonstrated the specific dendritic features and the distal axon origin of VENs, making them distinguishable from other modified pyramidal neurons (including oval or spindle-shaped bipolar neurons), most Golgi studies largely neglect these findings (Watson et al., 2006; Evrard et al., 2012; Butti et al., 2014; Correa-Júnior et al., 2020).

### Are VENs Present in Non-human Species?

It should be noted that in some non-primate species there are cells that on Nissl/anti-NeuN staining strongly resemble von Economo’s special cells and in some cases such cells were even demonstrated in the FI and ACC in the appropriate layer (Hof and van der Gucht, 2007; Hakeem et al., 2009). Nevertheless, there is still no data on the dendritic and axonal morphology of these cells in most non-primate species, which would help answer the question whether the cells described in non-primates are indeed the same type of specialized cells described by von Economo in humans. Moreover, even if a morphological correlate of VENs is found in non-primates, it should still be determined whether such cells are present only sporadically or they group in greater numbers in specific cortical regions, such as the FI and ACC.

In humans there is a clear grouping of specialized principal cells in the FI and ACC, however, we find it plausible that such or very similar cells could be found sporadically in other cortical regions, as we ourselves have described one such neuron found in BA9 of the PFC (Banovac et al., 2019). Nevertheless, during the last 30 years our research group analyzed Golgi sections from 47 infant and adult human specimens (Kostovic et al., 1991; Judaš et al., 2011) in 15 cortical regions, which include: the primary sensory cortex (Brodmann areas 3, 1, and 2), the primary motor cortex (BA4), the visual cortex (Brodmann areas 17, 18, and 19), the angular and supramarginal gyrus (Brodmann areas 39 and 40), the dorsolateral PFC (Brodmann areas 9 and 46), Broca’s area (Brodmann areas 44 and 45), the hippocampal formation and the entorhinal cortex (BA28). Detailed morphometric analysis was performed on around 800 cortical neurons, predominantly in the dorsolateral PFC, primary motor cortex and Broca’s area (Petanjek et al., 2008, 2011, 2019; Zeba et al., 2008; Sedmak et al., 2018; Banovac et al., 2019, 2020). During our research we have identified only a single neuron (outside the FI and ACC) on Golgi staining that has the dendritic and axonal morphology characteristic for von Economo’s specialized cells (Banovac et al., 2019). This suggests that even though VENs may be present in other human cortical regions, their number is disproportionately greater in the ACC and FI, which is supported even by studies that identified VENs/VEN-like cells in the human BA9 and BA10 using anti-NeuN staining (Fajardo et al., 2008; González-Acosta et al., 2018). Furthermore, even in the FI and ACC, there are morphological variants of cells with axonal morphology typical for von Economo’s specialized cells that, however, lack the typical cell body shape, thus they could easily be overlooked on Nissl and anti-NeuN staining. This means that one could easily conflate specialized cells with common spindle cells/spindle transformation, and vice-versa. Therefore, the identification of von Economo’s specialized cells in other cortical regions and non-primates should be done by demonstrating the dendritic and axonal morphology or by identifying specific markers or marker combinations that would enable the identification of VENs without relying solely on morphology.

### Perspective for Future VEN Research

The research on VENs is expanding and the fact that they have been described in numerous neuropathological conditions increases interest in studying these cells. However, some fundamental issues concerning VENs still appear to not have been completely resolved with different authors offering different and sometimes opposing interpretations of VENs. The lack of a consensus on basic terminology in the field reduces the reproducibility of studies because different authors do not use consistent definitions of VENs and use different methods for their identification in different species, cortical regions and layers. This makes drawing accurate conclusions on VENs extremely difficult and perpetuates speculative reasoning to explain inconsistencies between the findings of different studies.

Therefore, future VEN research should strive to resolve at least some of the following important issues:

(1) How should VENs be defined, which criteria should be used for their identification that would be consistent and applicable in different species, and which methodology should be the gold standard for confirming the presence of VENs in the brain?
(2) What are the functional properties of VENs and how is their unique morphology and cortical/laminar distribution tied to their neurophysiology?
(3) If VENs are projection neurons, where do they project and what are their targets? Do the projections of VENs differ from other projection neurons of the same layer and region or not? In which microcircuits of the brain are VENs involved?
(4) Why are VENs in humans abundantly present in only two cortical regions, while they appear to be ubiquitous in several completely unrelated species? Does the abundancy of VENs in specific cortical regions in the human brain have a practical significance for the functionality of the human cerebral cortex?

It is clear that attempting to address some of these issues will require developing new or expanding existing methods to better investigate VENs and their properties. If VENs are indeed a special type of neuron, as research strongly suggests being the case, at least in the human brain, their functional significance in humans should be the most important target of further research. Until VENs are clearly defined and their role in the functionality of the human cerebral cortex is well-understood, it is unlikely that it will be possible to fully appreciate the relevance of comparative and neuropathological studies in this field.

## Conclusion

To summarize, in this paper we gave a comprehensive overview on all available VEN research. Comparing the initial descriptions of VENs to modern research, we concluded that, for now, the most reliable way of identifying these cells is by visualizing their somato-dendritic morphology, which is still most commonly done on Golgi staining. Nevertheless, most research on VENs is still done on Nissl and anti-NeuN staining. Until specific molecular markers (or a specific combination of molecular markers) are identified, we propose that studies on VENs use additional methods (such as, but not necessarily limited to, Golgi staining) to confirm that the cells being studied are indeed VENs. This would significantly increase the robustness of the data in this field and would be particularly welcome in comparative studies. We found most studies gave little recognition to Cajal’s descriptions of the dendritic morphology and distant axon origin that clearly characterize VENs as a special cell type. Their distinct morphology combined with their abundancy in specific cortical regions suggest that they have important functional implications in the cortical areas they are found in. After a detailed review of all available literature on VENs, we consider that their presence outside the human FI and ACC is not clearly demonstrated, however, it is likely that VENs could be found sporadically in regions besides the human/primate FI and ACC (and possibly even in non-primates). Nevertheless, present evidence strongly suggests that VENs are abundant only in the FI and ACC of humans and almost certainly of other great apes, though the findings in other great apes should still be further verified by visualizing the cells using methods other than Nissl. Further morphological and functional research on VENs, using clear criteria for their identification, is needed to establish the functional and evolutionary significance of these cells.

## Author Contributions

IB and DS designed the study and analyzed the available literature. All authors drafted the manuscript and performed a critical revision of the manuscript.

## Conflict of Interest

The authors declare that the research was conducted in the absence of any commercial or financial relationships that could be construed as a potential conflict of interest.

## Publisher’s Note

All claims expressed in this article are solely those of the authors and do not necessarily represent those of their affiliated organizations, or those of the publisher, the editors and the reviewers. Any product that may be evaluated in this article, or claim that may be made by its manufacturer, is not guaranteed or endorsed by the publisher.
